# Investigation on dynamic mechanism of fault slip and casing deformation during multi-fracturing in shale gas wells

**DOI:** 10.1038/s41598-024-63923-x

**Published:** 2024-06-07

**Authors:** Chaojie Zhao, Yanxin Jin, Xue Wang

**Affiliations:** 1 Sinopec Research Institute of Safety Engineering Co., Ltd., Qingdao, 266000 China; 2 State Key Laboratory of Safety and Control for Chemicals, SINOPEC Research Institute of Safety Engineering Co., Ltd., Qingdao, 266000 China

**Keywords:** Casing deformation, Fault slip, Multi-fracturing, Dynamics mechanism, Petrology, Geodynamics

## Abstract

Fracturing horizontal well casing deformation has become very prominent, particularly in tectonic stress-concentrated shale gas fields, limiting the efficient development progress of shale gas. The main failure mode of casing shearing deformation had been attributed to fault slip caused by multi-fracturing. The current research did not provide a clear picture of the dynamic evolution relationship between hydraulic fracturing, fault slip, and casing deformation. In this paper, the dynamic model of fault slip induced by formation pressure change is established, incorporating the effects of stress drop, physical change of friction, and casing and cement-sheath resistance loads. The discontinuous displacement approach and explicit/implicit coupling iteration methods are used to reveal the relationships between the effective normal stress, shear stress, friction coefficient, and sliding velocity during the fault slip process. Furthermore, the microscopic process of casing deformation sheared by fault slip is investigated using static equilibrium theory, and a characterization method for determining the amount casing deformation caused by real-scale fault slip is proposed. The results show that three stages exist in the process of casing deformation sheared by fault slip, including trigger activation stage, accelerated slip stage, and deceleration slip stage. Fault slip is clearly influenced by fault strike. To reduce the amount of fault slip, the fault direction with the maximum in-situ stress should be avoided as much as possible. Serious casing deformation still occurs for large-scale activated faults even though the optimization measure of wellbore structure has been well taken. To fundamentally reduce the possibility of casing shear deformation, it is necessary to prevent fault slip through optimizing the design of hydraulic fracturing. This study lays the theoretical groundwork for the casing deformation control method in shale gas wells.

## Introduction

The mechanism of casing deformation during multi-fracturing in shale gas wells has emerged as a key topic of political and scientific discussion^1,2^, due to concerns that this event would seriously impede the efficiency and economic development of shale gas in China.

Many scholars have conducted extensive research on the main failure mode of casing deformation in fracturing wells, and the three main points are as follows. To begin, it is believed that the alternating temperature and Annulus Pressure Decrease (APD) effect produced during the fracturing process induces casing deformation and failure under extreme non-uniform load^3,4^. Adams^3^ and Sugden^4^ proposed the APD effect. It was stated that during hydraulic fracturing liquid channeling can disrupt the seal between the low permeability reservoir and casing. Furthermore, as hydraulic fracturing occurs, the temperature of the bound fluid that channels at the seal is reduced, the volume is significantly reduced, and the pressure drops rapidly, resulting in an imbalance of internal and external pressure of the casing and casing deformations. According to Yin et al.^5^, large-scale fracturing causes a rapid drop in wellbore temperature, resulting in thermal stress load on the casing and casing deformation. Tian et al.^6^ considered the alternating temperature and APD effect on the well during hydraulic fracturing, and that a pressure difference of 84 ~ 91 MPa formed between the inner and outer wall of the casing, significantly increasing the possibility of casing deformation. The second viewpoint focused on the extreme non-uniform disturbed stressnear the wellbore after hydraulic fracturing, which caused casing collapse and deformation. According to Zhang et al.^7^, the induced stress generated by hydraulic fracture increased the radial stress and the stress heterogeneity on the casing, resulting in an extremely increased possibility of casing failure. Furthermore, Lian et al.^8^ proposed a two-dimensional plane strain numerical model that used ABAQUS software to calculate the Mises stress and deformation characteristics of the casing after fracturing. It was thought that the reservoir rock gradually weakened and the extreme non-uniform stress disturbed, resulting in an obvious shear effect and casing deformation (Fig. 4). Third, it was assumed that hydraulic fracturing caused fault slip, which sheared the casing and caused deformation. Zhao et al.^9^, Dong et al.^10^, and Chen et al.^11^ investigated the location relationship between casing deformation and faults in the Weiyuan-Changning shale gas field. It was discovered that the casing deformation points were mostly consistent with naturally weak planes or fault-developed formations. According to Zoback et al.^12^ and Tong et al.^13^, the formation would inevitably move along the weak interface or fault plane (as shown in Fig. 5) due to asymmetric fractures and disturbed stress caused by hydraulic fracturing, causing the casing to deform and fail due to shear effects. According to Warpinski et al.^14^ and Chen et al.^15^, the fracturing fluid flowed to the fault plane or weak surface during the fracturing process, increasing pore pressure and decreasing effective stress. As the critical value has been reached, the fault slipped and sheared the casing, causing deformation. Monroe and Breyer^16^ and Liu et al.^17^ proposed that shear load caused by a fault or weak plane slipping was the key factor for casing deformation by comparing the calculation result of fault slip based on micro-seismic monitoring with the measured casing deformation result.

Furthermore, field statistical data shows that the fracturing well casing deformation mode in shale gas wells is mainly sheared deformation, and the deformation point location is highly coincident with the fault orientation, implying that the third failure mode of the casing is widely accepted. The variation of the local effective stress caused by hydraulic fracturing disrupts the fault plane's equilibrium state, which can be estimated using the Amontons-Coulomb friction laws. Unfortunately, the quantified characterization of the fault slip process remains ambiguous, which could provide the key theoretical basis for casing deformation control. Scholars have done little research on the quantitative characterization method of fault slip induced by hydraulic fracturing, focusing primarily on the two aspects listed below. Firstly, the experienced displacement of fault slip was calculated using the focal mechanism principle, which established a correlation model between fault slip and micro-seismic moment magnitude^17^. Second, a semi-analytic and analytical model of slip when a hydraulic fracture communicates with a fault is established^18–24^, which quantifies the magnitude of fault slip under static initial conditions. The preceding study focuses primarily on the calculation of fault slip displacement using static equilibrium theory, ignoring stress drop, the physical change of friction, and casing resistance loads during the fault slip process. It is difficult to determine the dynamic evolution of fault slip and casing deformation caused by hydraulic fracturing.

Therefore, in this work, the dynamic model of fault slip is established fully incorporating the stress release and physical change of friction during fault slip. The microscopic process of casing deformation sheared by fault slip is then investigated based on D'Alembert's principle, which establishes a transformation relationship between dynamic and static problems by adding inertial Force. Moreover, the proposed model is validated using an experimental approach and field case data. Furthermore, a sensitivity analysis of fault slip state and casing deformation are clarified, and fault slip-induced casing deformation control methods are extensively discussed by conducting the sensitive analysis.

## Methodology

### Dynamic mechanical model of fault slip

**Figure 1:**
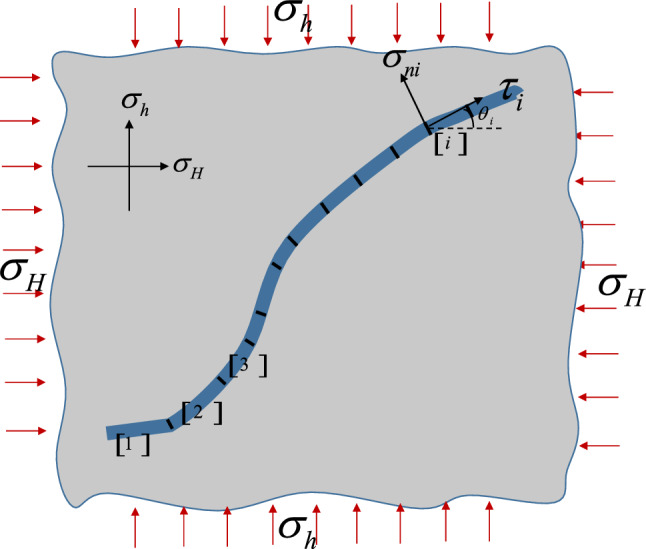
The fault stress diagram.

The fault stress is simplified to the mechanical model shown in Fig. 1. The infinite boundary is subject to the maximum and minimum horizontal stress of the formation, and the fault is subject to fluid pressure. The fault is composed of N units of small fault body, with slightly different strike.

Previous research has calculated the pore pressure during multi-fracturing^25,26^. It illustrates how well the pore pressure in the reservoir near the wellbore increases (even more than 20 MPa). Because of the high transmissibility of the fault plane, the pore pressure in the fault near the wellbore increases as well. According to the frictional equilibrium equation:

1 $$ \tau = S_{o} + \mu_{o} \sigma^{\prime}_{ni} $$

where, $\sigma^{\prime}_{ni}$ is the effective stress, which is defined as:

2 $$ \sigma^{\prime}_{ni} = \sigma_{ni} - P_{i} $$

The shear stress $\tau$ and principal stress $\sigma_{n}$ of the fault plane can be calculated from the boundary geo-stress conditions through the coordinate transformation. The transformation formula is as follows:

3 $$ \begin{aligned} \sigma_{ni} & = \sigma_{h} \cos^{2} \theta_{i} + \sigma_{H} \sin^{2} \theta_{i} \\ \tau_{i} & = \left( {\sigma_{H} - \sigma_{h} } \right)\cos \theta_{i} \sin \theta_{i} \\ \end{aligned} $$

The static friction stress on the fault plane is equal to the shear stress for a closed fault. When the shear stress exceeds the maximum static friction stress of the fault plane, the fault begins to slide. According to the dynamic theory proposed by Rice et al.^27^, the damping effect of inertia on the fault slip can be expressed as the product of the slip velocity and the damping coefficient when the shear stress and the friction resistance stress differ by a certain amount. Then, the dynamic equation of fault slip is expressed as:

4 $$ \tau_{i} - \eta v_{i} = \mu_{fi} \sigma^{\prime}_{ni} $$

where, $\mu_{fi}$ is the dynamic friction coefficient at fault plane unit *i*.

The sliding rock friction experiment demonstrates that the dynamic friction coefficient of rock varies with time, speed, and other parameters. As the two rock surfaces slide past each other, asperities that have been in contact will lose contact, and new asperities will come into contact. The meanlife time of asperity contact is roughly given by t ≈ d/v, where v is the relative velocity between the two surfaces, and d is a characteristic asperity diameter.

The relationship between dynamic friction coefficient and sliding speed is obtained:

5 $$ \mu_{fi} = \mu_{0} + a\ln \left( {1 + \left( {d/vt_{0} } \right)} \right) $$

According to the “rate/state” friction law when fault slip proposed by Segall^28^, the relation equation of dynamic friction coefficient is:

6 $$ \mu_{fi} = \mu_{0} + a\ln \frac{v}{{v_{0} }} + b\psi $$

where, *ψ* is the state variable, indicating the damage on the fault surface; *a, b* is dimensionless constant, reflecting the extent to which the friction coefficient changes with the change of velocity and fault plane state.

7 $$ \frac{d\psi }{{dt}} = - \left( {v/\lambda } \right)\left[ {\psi + \ln \left( {v/v_{0} } \right)} \right] $$

After the velocity is stable, the fault state variable is a constant value, implying $\frac{d\psi }{{dt}} = 0$, so $\psi = - \ln \left( {v/v_{0} } \right)$.

Equation (6) can be expressed as:

8 $$ \mu_{fi} = \mu_{0} + \left( {a - b} \right)\ln \left( {v/v_{0} } \right) $$

Incorporating the stress release effect during fault slip (Stress drop model, SDM), the local stress of the fault plane changes, which needs necessary modification.

For strike-slip fault, the variety of shear stress during fault slip is:

9 $$ \Delta \tau = \frac{2}{\pi }\frac{GD}{h} $$

For dip-slip fault, the variety of shear stress during fault slip is:

10 $$ \Delta \tau = \frac{{4\left( {\lambda + G} \right)}}{{\pi \left( {\lambda + 2G} \right)}}G\frac{D}{h} $$

where, $\Delta \tau$ is the difference between the far-field tectonic shear stress and the friction stress on the fault plane; *G* is the shear modulus, the average value of the crust is 33 GPa; *h* is the activation depth of the fault; *D* is the dynamic sliding displacement of the fault.

The change of interface normal stress caused by the release of fault slip stress is:

11 $$ \Delta \sigma_{nf} = \frac{\sin \theta \Delta \tau }{{\cos \theta }} $$

Simultaneously, it is assumed that the fault plane is an isotropic elastic body with a stress–strain relationship that follows Hooke’s law, namely:

12 $$ T = \frac{{2G\nu_{p} }}{{1 - 2\nu_{p} }}trace\left( \varepsilon \right)I + 2G\varepsilon $$

where, $\varepsilon$ is the strain tensor at each point on the fault plane; $I$ is the coefficient matrix^29^.

The cumulative slip displacement at any point on the fault plane is equal to the integral of the slip velocity at that point, namely:

13 $$ D_{c} = \int {vdt} $$

The spatial discretization scheme-displacement discontinuity method and time domain discrete method are applied for the model solution, which is shown in Appendix A and B, respectively.

### The mechanical analysis of casing deformation sheared by fault slip

Section "Dynamic mechanical model of fault slip" examines the dynamic change process of fault slip without taking into account the effect of the well element (casing cement sheath), while the process of well deformation sheared by fault dynamic slip is unclear. Based on the D’Alembert’s principle, the dynamic process of casing deformation caused by fault slip would be analyzed with static equilibrium method, referred to as dynamic-static method. In this section, the evolution of mechanical load and deformation of the well (casing and cement sheath) during fault slip have been analyzed, incorporating the effect of well resistance to deformation and inertial force.

**Figure 2:**
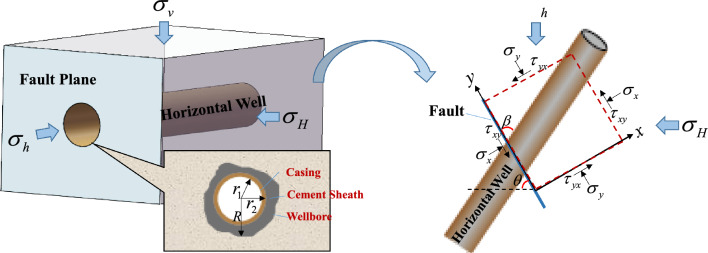
The representation model of flow conductivity in fracturing area nearby well.

For a strike-slip fault, the fault plane is parallel to the vertical stress direction. The force analysis between the fault plane and wellbore can be conducted at one plane (shown in Fig. 2) since the vertical stress component will not directly affect the fault slip.

The stress on the fault plane is calculated by positioning the y-axis parallel to the fault plane and the x-axis vertically to the fault plane. The geo-stress decomposition transformation expresses the fault plane's normal stress in the x-direction and shear stress in the y-direction as:

14 $$ \begin{gathered} \sigma_{x} = \sigma_{H} \sin^{2} \theta - \sigma_{h} \left( {\sin^{2} \theta - 1} \right) \hfill \\ \tau_{yx} = \left( {\sigma_{H} - \sigma_{h} } \right)\sin \theta \cos \theta \hfill \\ \end{gathered} $$

where, $\sigma_{x}$ is the normal stress on the fault plane, MPa; $\tau_{xy}$ is the shear stress on the fault plane, MPa; $\sigma_{H}$,$\sigma_{h}$ are the maximum and minimum horizontal in-situ stress, MPa; $\theta$ is the included angle between fault strike and maximum horizontal in-situ stress, °.

#### Before fault slip

During hydraulic fracturing, the fracturing fluid enters the fault plane. As a result, the fault plane is subjected to the contact normal stress of the formation as well as the fracturing fluid pressure in the perpendicular direction. The effective normal stress on the fault plane is expressed as:

15 $$ \sigma_{n} = \sigma_{x} - p_{f} $$

Before being activated, the fault is subject to static friction and possible shear stress of the shaft against sliding. Based on the balance theory, the mechanical balance relationship along the fault plane can be established:

16 $$ \sigma_{{\text{y}}} A_{{\text{f}}} + \tau_{{{\text{xy}}}} A_{{\text{f}}} = \sigma_{{\text{y}}} A_{{\text{f}}} + f + \tau_{{\text{c}}} A_{{\text{c}}} $$

where, $A_{f}$ is the fault activation area, m^2^; $p_{f}$ is the formation pressure of fault plane, MPa; $\sigma_{n}$ is the effective normal stress of the fault plane, MPa; *f* is the static friction force of fault plane, $f = \mu_{s} \sigma_{n} A_{f}$, 10^6^N; $\tau_{{\text{c}}}$ is the shaft shear stress, MPa; $A_{{\text{c}}}$ is the effective stress area of the shaft on the fault plane (the casing and cement sheath form an elliptical ring, which is related to the included angle $\beta$), m^2^; $\mu_{s}$ is the static friction coefficient of the fault plane.

#### After fault slip

When a fault slips, static friction on the fault plane undergoes dynamic friction, and the friction coefficient differs with slip speed. The expression of dynamic friction is as follows:

17 $$ f_{d} { = }\mu_{d} \sigma_{{\text{n}}} A_{{\text{f}}} $$

where, $\mu_{d}$ is the dynamic friction coefficient of the fault plane.

The shear stress load acting on the shaft section can be expressed as:

18 $$ \tau_{w} = \left\{ {\begin{array}{*{20}c} {\frac{{\tau_{{{\text{xy}}}} A_{{\text{f}}} - f_{d} }}{{A_{{\text{c}}} }}\left( {v = 0} \right)} \\ {\frac{{\tau_{{{\text{xy}}}} A_{{\text{f}}} - f_{d} - \eta v}}{{A_{{\text{c}}} }}\left( {v \ne 0} \right)} \\ \end{array} } \right. $$

The process of well sheared deformation is divided into two stages: integrity cement sheath and failure cement sheath. When the cement sheath retains its integrity, the total resistant load for well shearing is primarily generated by the cement sheath and casing. Because of the cement sheath's low shearing strength, it is easily damaged when the fault slips. The total resistant load for well shearing generated solely by casing deformation is then equal to the cross-sectional area of the casing. The shear stress of the casing is:

19 $$ \tau_{{\text{c}}} = \left\{ {\begin{array}{*{20}c} {E\varepsilon^{e} + \lambda E\varepsilon^{p} ,\left( {v \ge 0} \right)} \\ {\frac{{\tau_{{{\text{xy}}}} A_{{\text{f}}} - f_{d} }}{{A_{{\text{c}}} }},\left( {v = 0} \right)} \\ \end{array} } \right. $$

where, $\varepsilon^{e}$ is the elastic strain of casing; $\varepsilon^{p}$ is the plastic strain of the casing; $E$ is the elastic modulus of the casing; $\lambda$ is the plastic strain coefficient of the casing.

When the effective shear force (after overcoming the friction) on the well generated by fault sliding overcomes the shear resistance of the casing, the casing will slip and deform with the fault until the two forces are balanced. Finally, the fault slip and casing deformation ends at the wellbore interface. Because the slip of the remote fault continues, the deformation of the casing is not equal to the total slip of the fault.

## Results and discussion

### Validation and analysis of the dynamic model of fault slip

**Figure 3:**
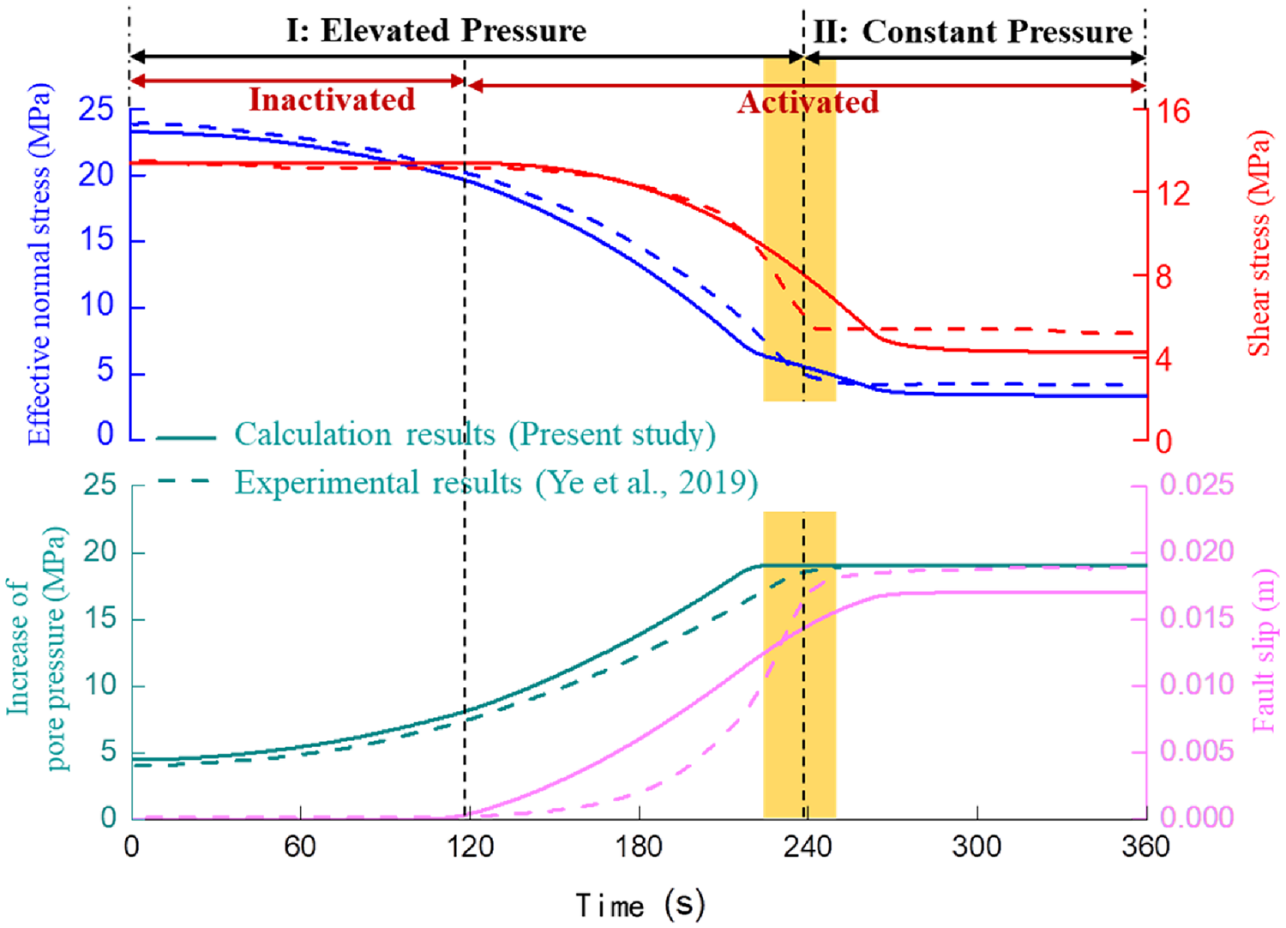
The dynamic response process of fault slip induced by pore pressure variation.

This paper utilizes the slip experiment results of artificial fractures on shale cores from the Eagle Ford field^30^ as an example to perform model calculation and verification. The “Matlab” software is employed (URL: http://www.mathworks.com, Version number: Matlab R2016a) to realize the calculation. Table 1 lists the relevant experimental condition parameters as well as the parameters required for model calculation. The model calculation results and the shear slip test results are shown in Fig. 3.

**Table 1:** The mechanical properties and geometric parameters of fault.

Dynamic calculation model		Slipping experimental^30^	
Parameter	Value	Parameter	Value
Max horizontal in-site stress (MPa)	51	Axial stress (MPa)	51
Min horizontal in-site stress (MPa)	20	Confining pressure (MPa)	20
Initial pore pressure (MPa)	3.5	Initial pore pressure (MPa)	3.5
Pore pressure after fracturing (MPa)	19.05	Maximum injection pressure (psi)	2900
Static friction coefficient	0.59	Initial friction coefficient of sample	0.59
Shear modulus of shale (GPa)	10	Shear modulus of sample (GPa)	10
Fault orientation (°)	30	Angle between fracture and axial direction (°)	30
Fault type	strike	Length of sample (inch)	3.1
Vertical length of fault (m)	100	Rock sample diameter (inch)	1
Initial slip velocity (m/s)	10–6	Loading rate (m/s)	10–6
Speed influence coefficient a	0.011		
State influence coefficient b	0.014		
Friction damping coefficient (MPa/(m/s))	20

There are two stages of pore pressure: an elevated pressure stage and a constant pressure stage. Here, “elevated pressure” means the gradually increasing injection pressure of fracturing fluid in the fault plane; the “constant pressure” means the injection pressure that is held as constant after it reaches the target. As shown in Fig. 3, both the model calculation results and the experimental measurement results show that the cumulative slip distance of the fault is 0.018 m, accompanied by the release of 20 MPa normal stress drop and 9 MPa shear stress drop, and the overall law of the two is similar. Moreover, because the calculation model assumes that the fault plane has uniform lithology, and the actual rock sample cannot achieve complete uniformity, when the pore pressure reaches its maximum value, the fault slip abruptly jumps, resulting in a sharp change in displacement. Overall, the calculation results of the model established in this paper are essentially consistent with the experimental measurement results (the difference is about 8.4%, which is within the allowable range), confirming the model's reliability.

**Figure 4:**
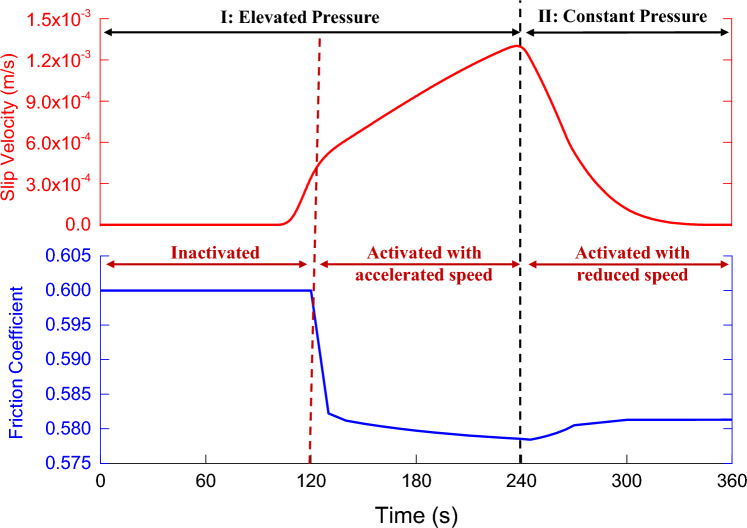
The variation of friction coefficient and slip velocity during fault slip.

As shown in Fig. 4, there are three stages in the process of fault slip induced by formation pressure according to the fault slip state.

Triggering activation stage (the first stage): The effective principal stress (solid blue line) at the fault plane decreases (with a decrease of about 4 MPa), as the formation pressure (solid green line) at the fault plane continues to rise. While the shear stress at the fault plane remains stable, the fault state remains stable, and thus the fault slip amount (pink line) is zero.

Accelerated slip stage (the second stage): When the formation pressure is increased to 8.2 MPa, the fault's stress balance is broken. The initial velocity slip is 10^–6^ m/s, and the friction coefficient of the fault plane suddenly decreases. The slip velocity then increases (from 10^–6^ m/s to 10^–3^ m/s). Furthermore, as energy is released, the effective normal stress and shear stress of the fault plane decrease. And the cumulative displacement of the fault slip is 0.015 m.

Deceleration slip stage (the third stage): As the friction coefficient of the fault plane increases, the slip velocity decreases. Besides that, the fault plane's normal stress and shear stress continue to decrease (the "stress drop" does not exceed 1 MPa), until the fault plane's shear stress and maximum static friction reach a new balance. Finally, the slip velocity drops to zero, and the slip ends. While comparing the second stage and third stage of the fault slip process, it's also discovered that the energy release and accumulated slip amount of the fault slip occur mainly in the accelerated slip stage.

### The dynamic process of casing deformation based on field case

**Figure 5:**
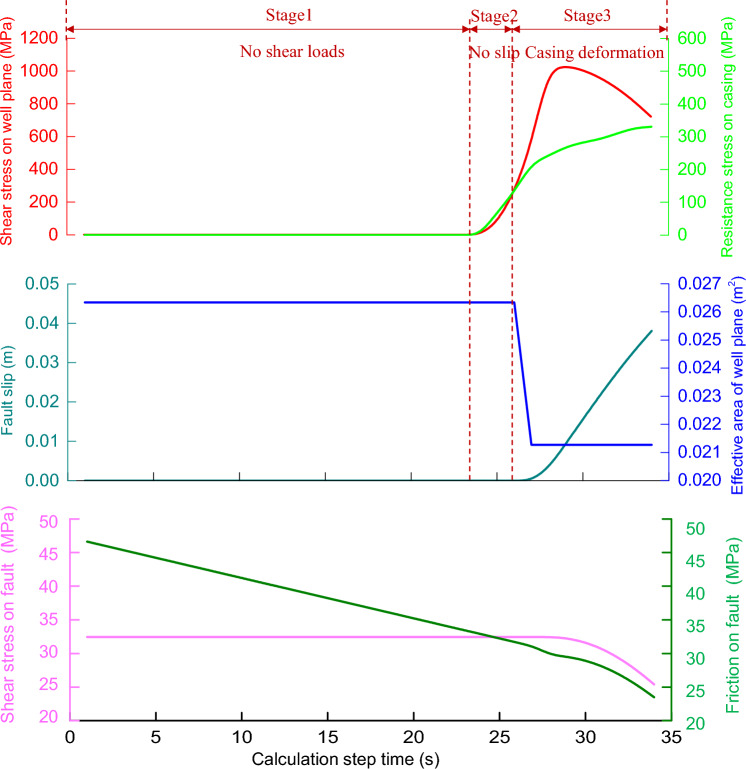
The evolution of casing deformation under shearing loads caused by fault slip.

To use the case of casing deformation caused by fault slip in Changning HXX as an example, the dynamic shearing process and deformation amount of casing during fault slip are calculated and analyzed using the model developed in this paper. The results are shown in Fig. 5. The parameters of the well (casing cement sheath) are shown in Table 2. The casing shear stress–strain curve is derived from the indoor full-size casing shearing experimental test.

**Table 2:** The geometry and material parameters of the casing-cement sheath in Well Changning HXX.

Well element	Grade	Internal diameter (mm)	Thickness (mm)	Elastic modulus (GPa)	Poisson's ratio
Casing	V110	113.3	14.2	207	0.26
Cement sheath	N/A	139.7	38.1	10	0.25

As shown in Fig. 5, there are three stages in the deformation process of fault slip shearing casing. In the stage 1, as the formation pressure rises, the effective normal stress and maximum static friction force of the fault plane decline, while the fault shear stress is less than the maximum static friction stress of the fault plane, resulting in a shear load acting on the wellbore of 0 at this stage. It implies that there are no shear resistance loads occurring on the casing. Furthermore, because the cement sheath is intact during the process, the effective section of the wellbore is the sectional area of the combination of cement sheath and casing, with an area of 0.026 m^2^.

In the stage 2, the fault shear stress exceeds the faults plane’s maximum static friction stress, and the shaft begins to generate a shear load. Because the shear strength of the cement sheath is insufficient (in comparison to the casing), it fails at the initial stage. The effective section area of the wellbore is reduced to 0.021 m^2^ (the casing section area), and the shear load of the wellbore is carried solely by the casing.

In the stage 3, the fault starts to slip once the shear load of the fault acting on the casing exceeds the sum of the static friction load of the fault plane and the shear load of the casing. As a result, with the same displacement, the casing generates large shear deformation, implying a large plastic deformation stage. At this point, the shear deformation constitutive relationship of the casing determines the maximum shear resistance of the casing. While the difference between the shear force load at the fault interface and the friction load (the effective shear force acting on the shaft) is significantly greater than the shear resistance load of the casing, the fault accelerates sliding. During the process of fault sliding, the dynamic friction coefficient decreases; on the other hand, the stress drop occurs, as the fault plane continuously releases energy. As a result, the stress at the fault plane begins to balance, and the sliding speed declines. At the same time, as the deformation increases, the shear load of the casing increases. Finally, the fault-wellbore unit is statically balanced, and the casing deformation ends.

**Figure 6:**
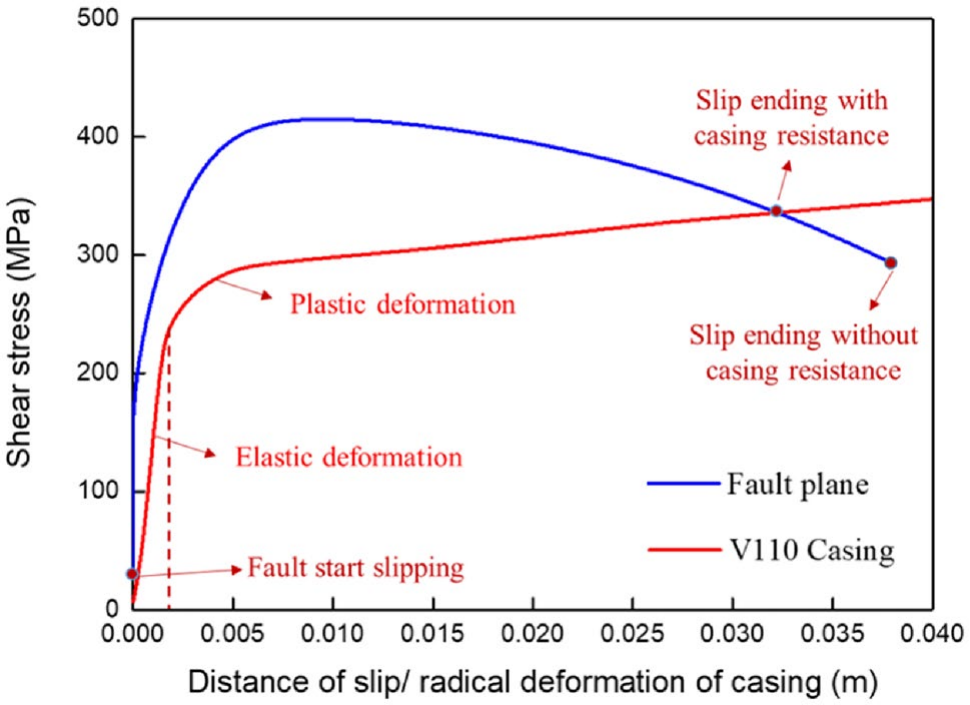
The scale of casing deformation under fault slip.

As shown in Fig. 6, the fault slip at the wellbore interface is 31 mm considering the effect of well (casing-cement) resistance, while the fault slips at the distance is 38 mm neglecting the effect of well (casing-cement) resistance. It indicates that the effect of well (casing-cement) resistance cannot be directly ignored.

Moreover, the calculation result of casing deformation is 31 mm, while the measurement result by the multi-arm caliper is about 27.66 mm. The difference (3.4 mm) between the calculation and measurement results is within the acceptable range in engineering, which verifies the reliability of the calculation model.

### Sensitivity analysis for casing deformation

A variety of factors, including fault type, formation pressure variation, and fault strike, impact casing deformation caused by fault slip during hydraulic fracturing. The effects of the above parameters on casing deformation caused by fault slip are investigated using the proposed model in this paper. The sensitivity analysis aims to find the best control method for fault slip and casing deformation in shale gas wells.

#### Fault type

**Figure 7:**
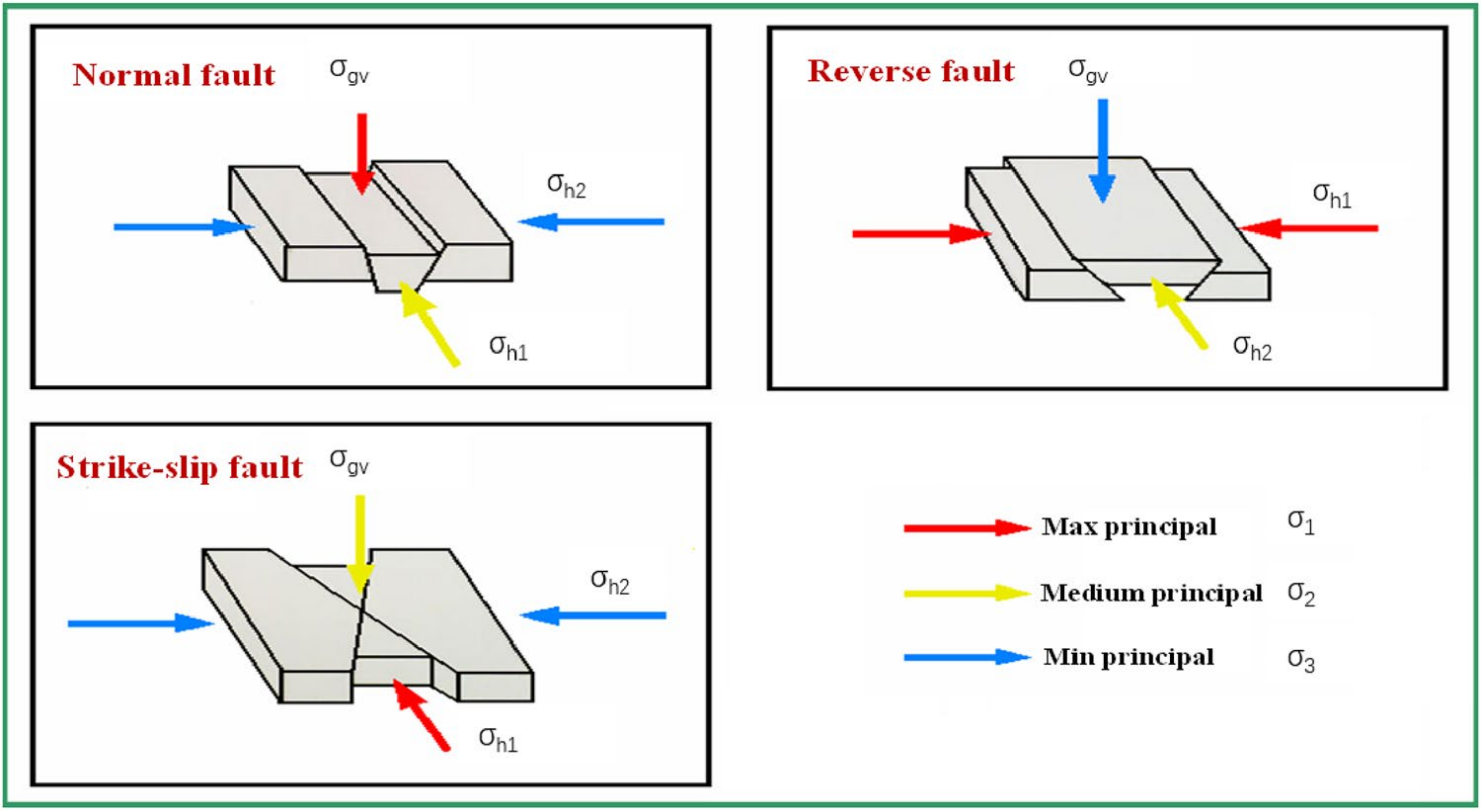
The diagram of the bearing model for different types of faults.

**Figure 8:**
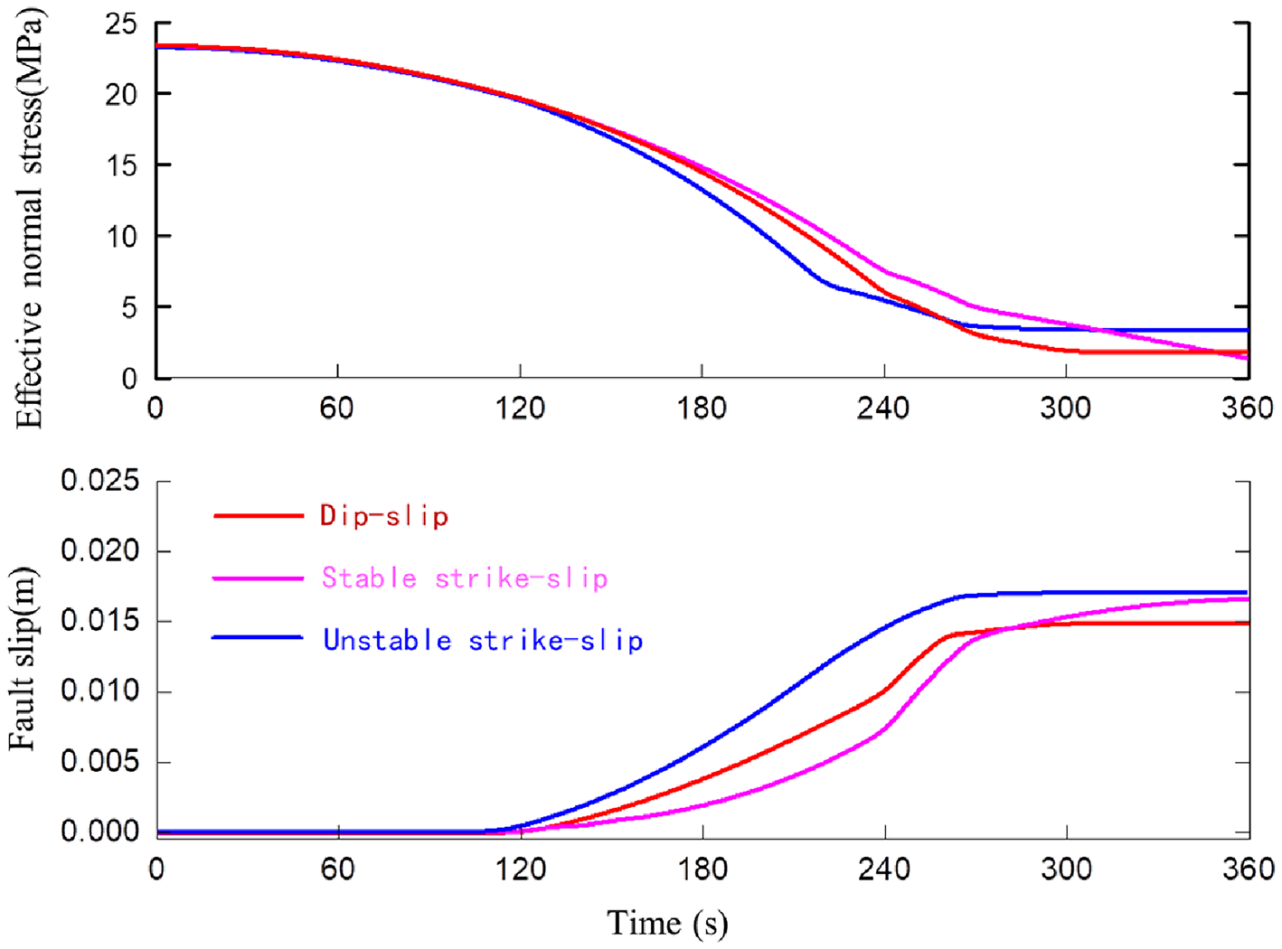
The variation of slippage and normal stress during fault slip for different faults.

**Figure 9:**
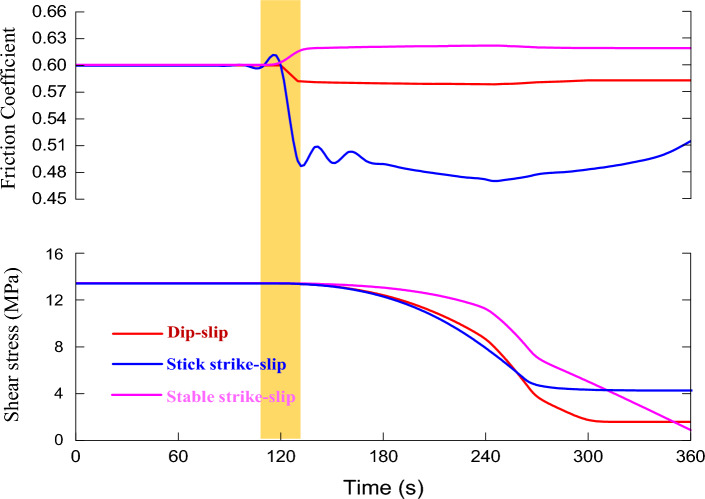
The variation of dynamic friction coefficient and shear stress for different types faults.

According to the relationship of fault slip position, it can be divided into a normal fault, reverse fault, and strike-slip fault. Anderson’s theory describes the relationship between fault principal stress and different types of faults (as shown in Fig. 7). Normal and reverse faults are both in dip-slip mode, which means their slip moves vertically along the fault plane; strike-slip faults move horizontally along the fault plane. According to the slip state of its section, there are two types of sliding for a strike-slip fault: stable-sliding and stick–slip. The former is stable- sliding, while the latter occurs rapidly and causes earthquakes. The former is stable sliding, while the latter is rapid and causes earthquakes. The mineral properties of the fault plane influence the properties of stable-slip and stick–slip. If a < b, an unstable slip occurs, referring to stick–slip. If a > b, the sliding is stable. Table 3 shows the various types of fault parameters used in the calculation, and the results are shown in Figs. 8 and 9.

**Table 3:** The mechanic’s properties and geometric parameters of different faults.

Fault type	In-site stress			Lithology	
	Vertical stress (MPa)	Maximum horizontal stress (MPa)	Minimum horizontal stress (MPa)	a	b
Dip-slip fault	51	34	20	0.014	0.011
Stick strike-slip	34	51	20	0.014	0.011
Stable strike-slip	34	51	20	0.011	0.014

Figure 8 shows that, the cumulative slip amount of the dip-slip fault is less than the cumulative slip amount of the strike-slip fault under the same formation pressure change. While the main stress and shear stress at the fault interface decrease more. It implies that the shear displacement load of a strike-slip fault on the wellbore (casing) is greater than that of a dip-slip fault under the same conditions. The majority of faults in the Sichuan Basin shale formation are strike-slip faults, which will aggravate the degree of casing deformation. It is consistent with the phenomenon of severe casing deformation of shale gas wells in this area.

As shown in Fig. 9, the dynamic friction coefficient of the stable strike-slip fault differs significantly from that of the stick strike-slip fault. For the stable slip fault (pink curve), its fault interface coefficient a > b. After the fault is activated and slipped, the dynamic friction coefficient of the fault surface gradually increases, and the slip time is long, with no significant energy release. For stick–slip fault (blue curve), its fault interface coefficient a < b, that is, the interface is brittle and easy to be broken after the fault is activated, and the previously contacted micro-convex body will lose contact, while the new micro-convex body will have a contact, so its dynamic friction coefficient fluctuates during the fault sliding process; After that, the dynamic friction coefficient drops sharply due to the influence of sliding speed, and is accompanied by obvious energy release.

There is no noticeable difference in the cumulative slip amount between the stick–slip and strike-slip faults, indicating that the shear displacement load of the shaft is not different between the two. That is, stable slip type fault slip can also cause serious shear deformation of the wellbore, but it will not release energy rapidly and will not be monitored by micro-seismic events, which explains some casing deformation events during hydraulic fracturing of shale gas wells where no micro-seismic signals are monitored.

#### Variety of formation pressure

**Figure 10:**
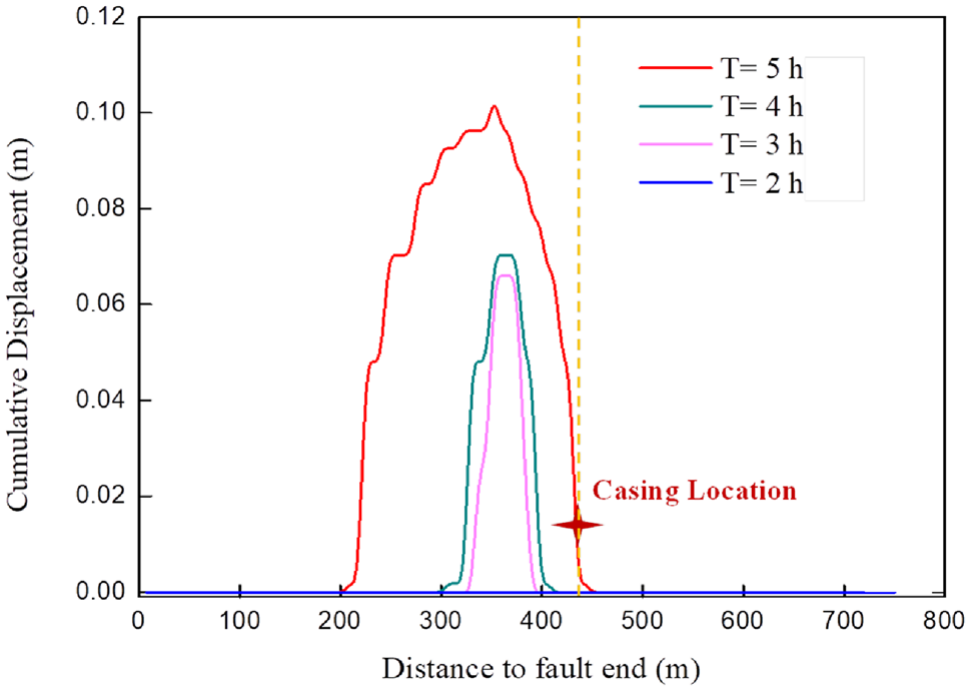
The evolution of fault slip during fracturing in stage 5.

**Figure 11:**
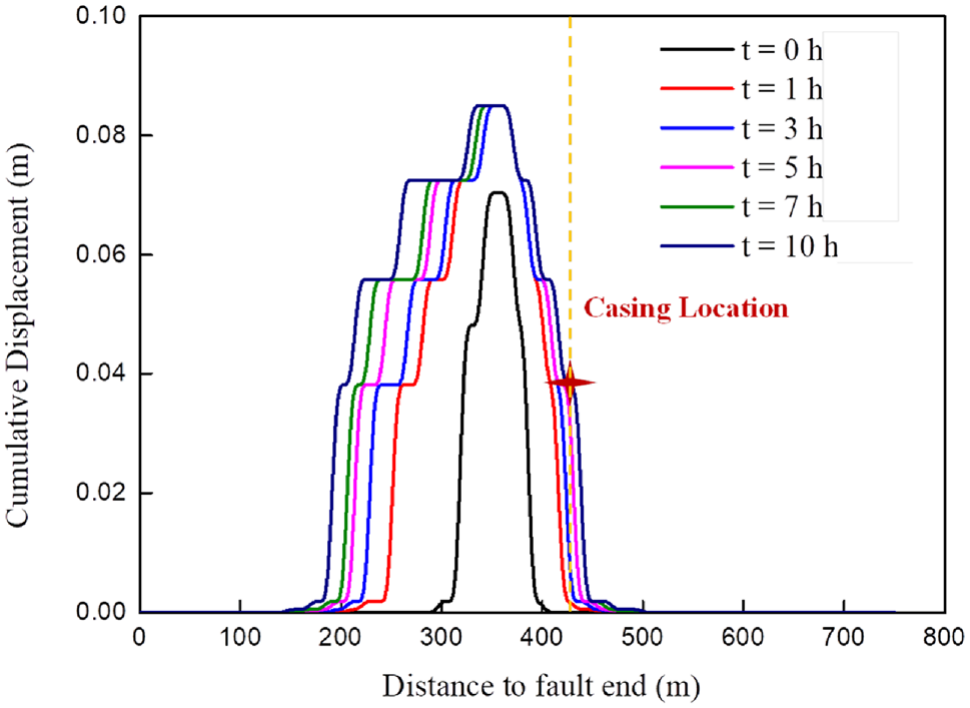
The evolution of fault slip after fracturing in stage 5.

During multi-stage fracturing, the formation pressure at the discontinuity interface changes dynamically with fracturing time, affecting the fault slip state. The fault slip along with hydraulic fracturing is analyzed to reveal the evolution of interrupted layer slip with the change of formation pressure during multistage fracturing, based on the result of a variety of formation pressure for the fault during the fifth fracturing stage in the Wei-H well^25^. The results are shown in Figs. 10 and 11.

##### During fracturing

As shown in Fig. 10, the fault’s stable slip was zero during the first two hours. The activation area is the fault section at 330 m-390 m when fracturing for 3 h, and the maximum slip is about 6 cm, which is about 60 m away from the wellbore. The fault at the wellbore is still stable after 4 h of fracturing, and it will slip about 2 cm after 5 h of fracturing. According to the numerical model of fault slip and calculation conclusion of casing deformation^31^, its casing shear deformation can be almost ignored. However, there was an abnormality, that is, casing deformation, during the process of drilling the bridge plug after fracturing the fifth section of the well, which was inconsistent with the calculation results. The micro-seismic monitoring shows that many events are still monitored after the fracturing pump is stopped, implying that the fault slip continues after the fracturing pump is stopped.

##### After fracturing

When the fracturing pump is turned off for an hour, the activation length of the fault plane continues to expand (about 130 m) and the maximum cumulative slip increases from 7 cm to 8.5 cm, as shown in Fig. 11. Later, the formation pressure in the hydraulic fracture area tends to be consistent with the formation pressure at the fault plane, the fracturing fluid will no longer enter the fault zone, and the maximum accumulated slip of the fault will not increase further; however, the formation pressure at the fault plane remains uneven, and the fracturing fluid will continue to spread from the high-pressure area to the low-pressure area along the fault plane, extending the fault activation to both ends and increasing the fault’s accumulated slip amount. However, as the fracturing fluid diffusion breaks down, the fault activation extension ceases, and the fault activation length increases by less than 20 m between 7 and 10 h after the pump is stopped. After 10 h of pump stop, the slip of the fault near the wellbore increases to nearly 4 cm, which is enough to cause 20 mm-30 mm shear deformation of the casing and interfere with the passage of downhole tools. It explains why the casing deformation is found during the tool running process after the fracturing of the fifth section, rather than during the fracturing process.

#### Fault strike

**Figure 12:**
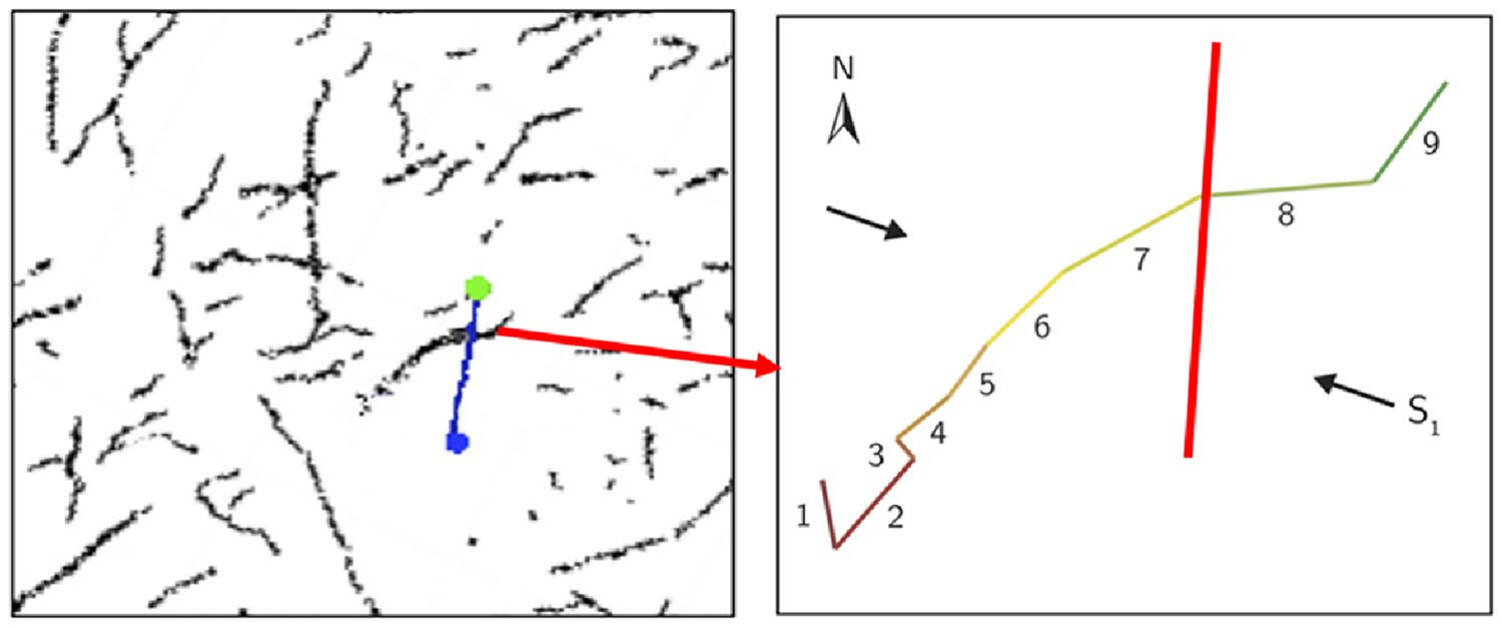
The sketch map of the position of strike-slip faults and well.

To determine the influence of fault strike on fault slip, a fault in a shale gas block in Changning, Sichuan Basin, will be used as an example. Figure 12 illustrates the seismic ant body identification map of a fault zone within a block. The green dot represents horizontal well A, the blue dot represents horizontal well B, and the blue line represents the horizontal well. The block is in a strike-slip fault stress state, with a maximum horizontal principal stress orientation of 110° and a formation pressure of 15 MPa. The fault continues to run through the horizontal shaft, which has an overall orientation of 40°–60°, but the orientations of each section differ. The marks of each section are shown in Fig. 12, and the specific orientations are shown in Table 4. The shaft passes through the junction of faults #7 and #8.

**Table 4:** Parameters of fault orientation.

No	Angle with maximum horizontal stress/°	Azimuth/°	Maximum horizontal in-site stress/MPa	Minimum horizontal in-site stress /MPa	Pore pressure increase value required for slip/MPa
#1	50	350	50	26.5	6.00
#2	80	30	50	26.5	27.59
#3	30	320	50	26.5	0.42
#4	60	50	50	26.5	9.17
#5	65	45	50	26.5	14.80
#6	75	35	50	26.5	23.63
#7	50	60	50	26.5	6.00
#8	25	85	50	26.5	0.70
#9	70	40	50	26.5	19.66

**Figure 13:**
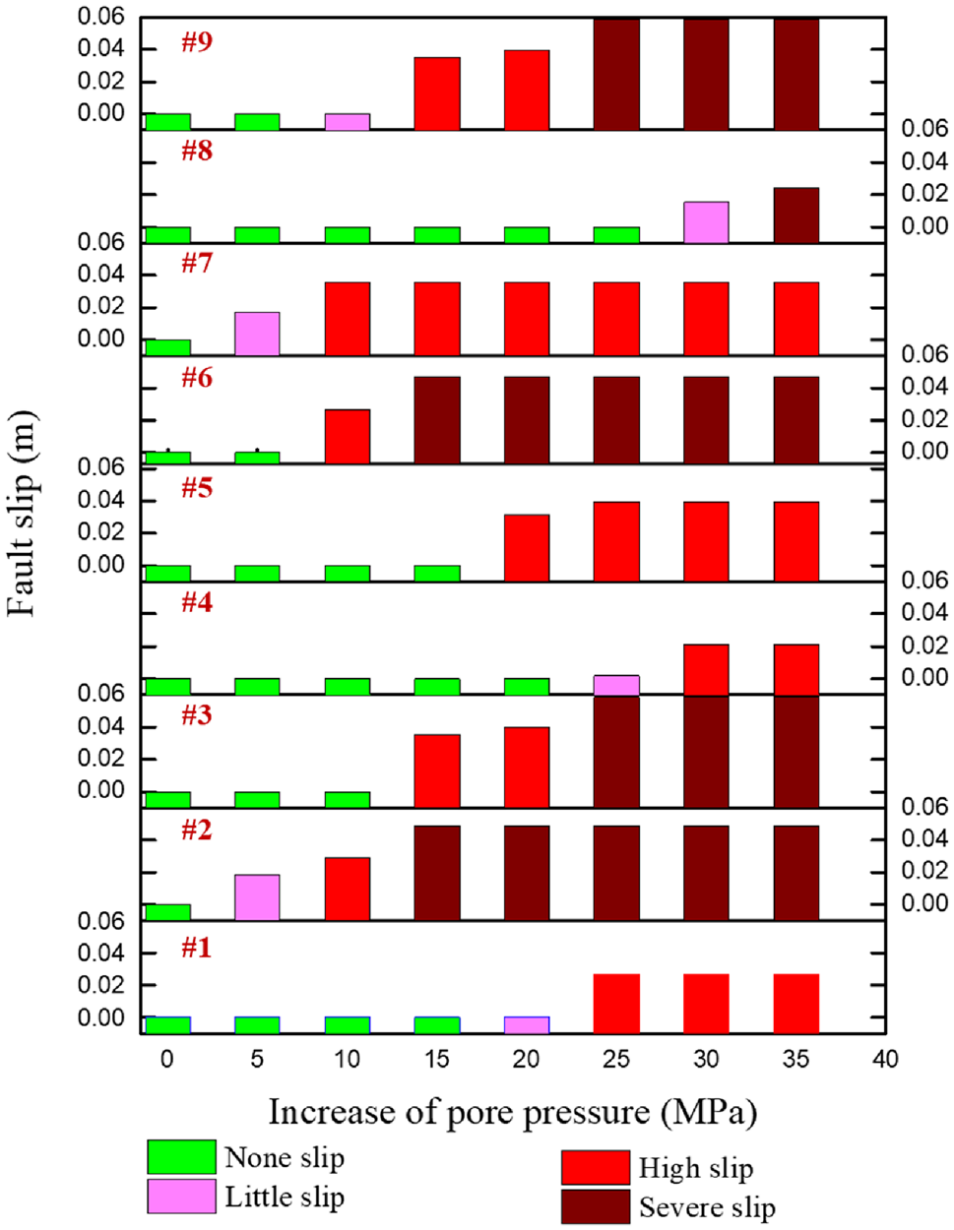
The variation of slip with varying formation pressure for different direction faults.

Figure 13 shows the change of the fault slip under different formation pressure increments. The green column in the figure represents that the fault is in a stable state and does not slip. The pink column indicates that the fault slip amount is less than 0.02 m, which has little impact on shaft deformation with slight slip. The red column indicates that the fault slip is 0.02 m-0.04 m, which will cause an obvious deformation of the wellbore and hinder the downhole tool from running in, with the medium slip. The dark red column indicates that the fault slip is greater than 0.04 m, resulting in severe wellbore deformation (casing diameter exceeds 20 mm) and serious slip.

When the local formation pressure increment exceeds 25 MPa (close to the minimum horizontal in-site stress), faults at #7 and #8 are in a serious slip, and irreversible deformation occurs at the real wellbore. However, faults #2, #6, and #9 are in non-slip or have a slight-slip state. If the wellbore is located near these positions, serious casing deformation will not occur. Even if they are located in the same block and have different orientations, the slip amount of each fault varies greatly. During drilling design, the critical factor of fault strike should be considered, particularly the fault location with too small a strike and the maximum in-site direction. Wellbore deformation caused by long distance fault slip should be avoided by avoiding fault locations with an orientation of 60°—110° in this area.

### The control method of casing deformation

At present, many measures have been taken to improve the casing steel grade and increase the casing wall thickness in response to the casing deformation problem of shale gas wells in the Sichuan Basin, but the casing deformation problem has not been significantly improved. Based on this, in conjunction with the established model and the fault and wellbore parameters of Well Ning-xx, the impact of casing type, well diameter size, thickening method of casing wall thickness, and other measures on reducing casing deformation is examined, providing a theoretical basis for the optimization analysis of on-site measures.

#### The optimization of casing type

**Figure 14:**
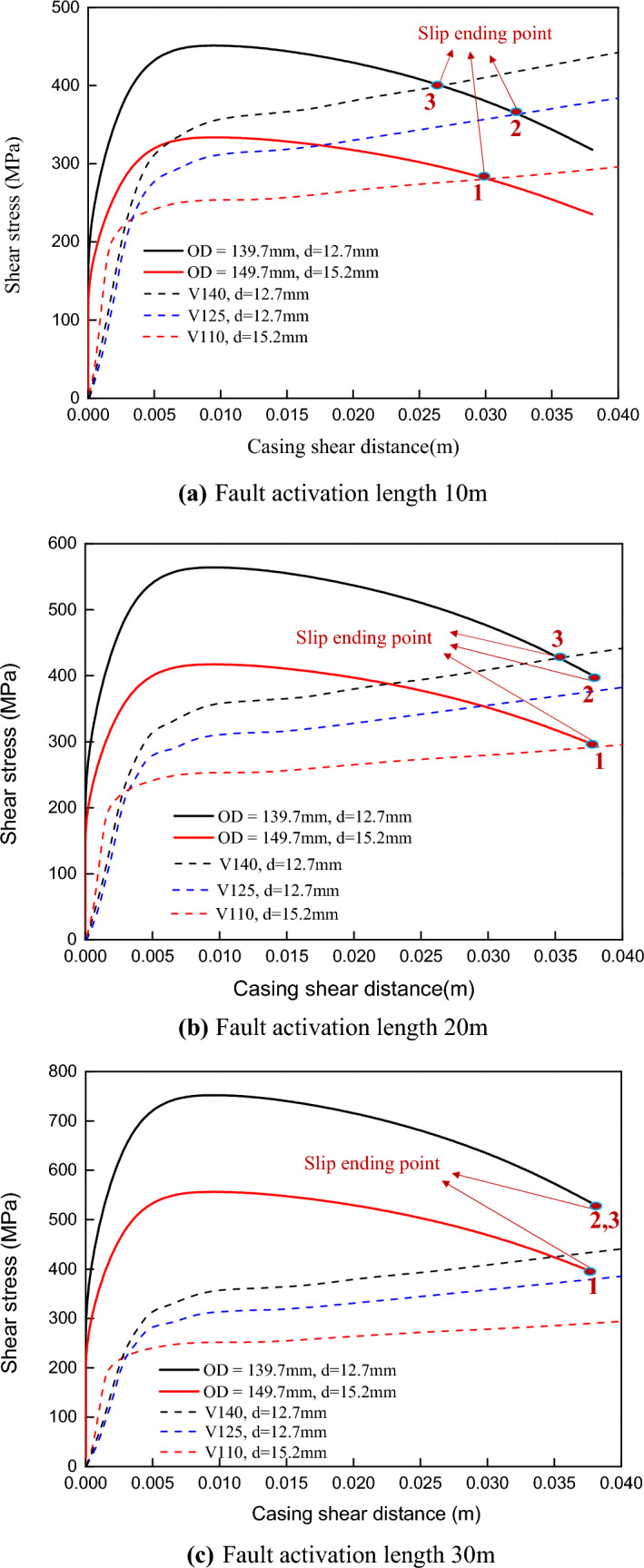
The deformation of different casings caused by fault slip.

As shown in Fig. 14a, when the fault excitation length is 10 m, the casing deformation can be reduced by increasing the casing strength or wall thickness. When the fault activation length is 20 m (shown in Fig. 14b), the casing wall thickness increases, but the casing deformation does not improve significantly (comparison points 1 and 2). Increasing the wall thickness can improve the shear strength of the casing but it also increases the outer diameter of the casing and the shear load acting on the shaft section, so the effect is not obvious. Point 3 is to improve the casing steel grade and slightly reduce the casing deformation.

As shown in Fig. 14c, when the fault activation length reaches 30 m, the deformation of the three types of the casing is the same (approximately equal to the fault slip). Increasing the casing wall thickness and steel grade can reduce casing deformation, but the effect is minor. However, the excitation length of the interruption layer in actual operation often reaches the level of 100 m, and the effect of improving the casing wall thickness and steel grade is negligible.

#### The design of well size

**Figure 15:**
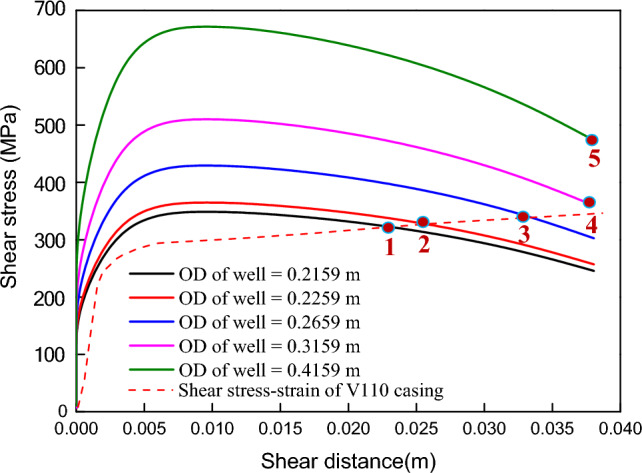
The scale of casing deformation under different well diameter.

As shown in Fig. 15, when the original diameter of the well (21.59 cm) is increased by 1 cm, the deformation of the casing along the fault plane increases from 23 to 26 mm. While the well diameter increases by 5 cm, the casing deformation along the fault plane reaches 34 mm. After the well diameter increased by more than 10 cm, the deformation of the casing along the fault plane is consistent with the fault slip without considering the shaft resistance load into account. It can be inferred that, under the same conditions, increasing the hole size increases the shear load and casing deformation of the sliding fault acting on the casing section. Under the same conditions, properly reducing the hole size is beneficial to reducing casing deformation caused by fault slip.

#### Thickening method of the casing wall

**Figure 16:**
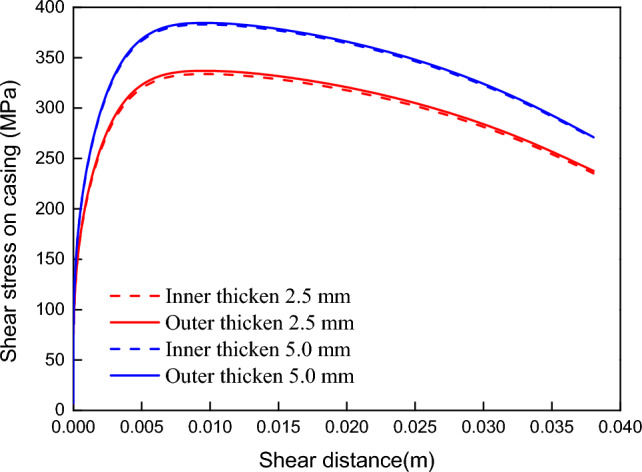
The shear stress on casing for different casing thicken methods.

In shale gas wells, the thickened casing is used as a important control method for preventing casing deformation. There are two ways to thicken the casing internally and externally. As shown in Fig. 16, the shear load of the externally thickened casing and the internally thickened casing of the fault is not significantly different (the shear load of the internally thickened casing is slightly lower) under the same thick-wall casing. It implies that the casing thickening method has no significant effect on reducing casing deformation caused by fault slip because it does not affect the shear load on the casing.

## Conclusions

This study establishes a comprehensive analytical model for calculating casing deformation caused by fault slip during multi-fracturing. The following conclusions were drawn from the field case study and model analysis.

1. The fault slip shear casing deformation process is divided into three stages: Trigger activation stage: the formation pressure rises, the effective principal stress at the fault plane continuously decreases, the stress at the fault plane is unbalanced, and the casing does not bear the shear force. Accelerated slip stage: friction coefficient decreases, slip speed increases, energy is released, effective normal stress and shear stress decrease, and casing plastic deformation occurs. Deceleration slip stage: the friction coefficient increases, the dynamic friction force, and the casing shear resistance exceed the fault shear force, the slip speed falls to zero, and the slip ends.
2. Under the same change of formation pressure, the cumulative slip of the dip-slip fault is less than that of the strike-slip fault; however, there is no discernible difference in the final cumulative slip between the stick–slip fault and stable-slip fault, indicating that both have a significant impact on casing deformation. However, the former has a fast-sliding speed and is accompanied by an energy release, whereas the latter has a slow sliding speed and no large energy release, which better explains the phenomenon that some casing change points in shale gas wells have not detected large-scale micro-seismic events.
3. After the fracturing pump is stopped, the fracturing fluid in the fault plane continues to diffuse from the high-pressure zone to the low-pressure zone along the fault plane, the fault activation length expands, and the active slip at both ends of the fault increase, which explains why casing deformation is more likely to occur.
4. Fault slip is clearly influenced by fault strike. To reduce the amount of fault slip, the fault direction with the maximum in-site stress should be avoided as much as possible.
5. To address the issue of fault slip shear wellbore deformation, when the fault activation scale is small, casing deformation can be improved by optimizing the wellbore structure (increasing the casing wall thickness and steel grade, decreasing the borehole size, and increasing the annulus deformable allowance). Serious casing deformation still occurs for large-scale activated faults due to the single optimization of wellbore structure. To fundamentally reduce the possibility of casing shear deformation, it is necessary to prevent fault slip through optimizing the design of hydraulic fracturing.

### Supplementary Information

Supplementary Information.

## Data Availability

Data is provided within the manuscript or supplementary information files.

## List of symbols

$K$: Transmissibility of formation
$\mu$: Viscosity of fluid
$k$: Permeability of formation
$h$: Thickness of formation
$\alpha$: Compressibility of coefficient of shale
$\beta$: Compressibility of coefficient of fluid
$\phi$: Porosity of formation
$Q$: The volume of injected fluid per unit of time
$$t$$: Fracturing time
$$p$$: Pore pressure
