# Supplementary material for: Investigation on dynamic mechanism of fault slip and casing deformation during multi-fracturing in shale gas wells
Source: Sci Rep. 2024 Jun 7;14:13164. doi: 10.1038/s41598-024-63923-x (PMC11161626; doi:10.1038/s41598-024-63923-x)
Supplement: Supplementary file 1 — Supplementary Information. [file 41598_2024_63923_MOESM1_ESM.docx]

**Appendix A**

As shown in Fig. 1, a segment of the continuous fault plane can be divided into N units of unit length. Furthermore, the mechanical equilibrium equation can be solved by using the linear elastic boundary element method proposed by Crouch and Starfield (1983).

Displacement discontinuity is defined as the displacement difference between two sides of the element:

$\begin{matrix} D_{x}=\mu_{x}\left(x,0_{-}\right)-\mu_{x}\left(x,0_{+}\right) \\ D_{x}=\mu_{y}\left(x,0_{-}\right)-\mu_{y}\left(x,0_{+}\right) \end{matrix}$ (A-1)

The displacement and stress of each element *i* caused by the constant displacement discontinuity $D_{x}^{j}$ and $D_{y}^{j}$ are expressed as follows:

$\begin{matrix} \mu_{s}=D_{s}^{j}\left[-\left(1-2\nu\right)\sin\beta_{_{i}}^{j}f_{_{,x}}^{j}+2\left(1-\nu\right)\cos\beta_{_{i}}^{j}f_{_{,y}}^{j}+y^{j}\left(\sin\beta_{_{i}}^{j}f_{_{,xy}}^{j}-\cos\beta_{_{i}}^{j}f_{_{,xx}}^{j}\right)\right] \\ +D_{n}^{j}\left[-\left(1-2\nu\right)\cos\beta_{_{i}}^{j}f_{_{,x}}^{j}-2\left(1-\nu\right)\sin\beta_{_{i}}^{j}f_{_{,y}}^{j}-y^{j}\left(\cos\beta_{_{i}}^{j}f_{_{,xy}}^{j}+\sin\beta_{_{i}}^{j}f_{_{,xx}}^{j}\right)\right] \end{matrix}$ (A-2)

$\begin{matrix} \mu_{n}=D_{s}^{j}\left[\left(1-2\nu\right)\cos\beta_{_{i}}^{j}f_{_{,x}}^{j}+2\left(1-\nu\right)\sin\beta_{_{i}}^{j}f_{_{,y}}^{j}-y^{j}\left(\cos\beta_{_{i}}^{j}f_{_{,xy}}^{j}+\sin\beta_{_{i}}^{j}f_{_{,xx}}^{j}\right)\right] \\ +D_{s}^{j}\left[-\left(1-2\nu\right)\sin\beta_{_{i}}^{j}f_{_{,x}}^{j}+2\left(1-\nu\right)\cos\beta_{_{i}}^{j}f_{_{,y}}^{j}-y^{j}\left(\sin\beta_{_{i}}^{j}f_{_{,xy}}^{j}-\cos\beta_{_{i}}^{j}f_{_{,xx}}^{j}\right)\right] \end{matrix}$ (A-3)

$\begin{matrix} \sigma_{n}=2G\left[2\sin^{2}\beta_{_{i}}^{j}D_{s}^{j}f_{_{,xy}}^{j}+\sin2\beta_{_{i}}^{j}D_{s}^{j}f_{_{,xx}}^{j}-D_{s}^{j}y^{j}\left(\cos2\beta_{_{i}}^{j}f_{_{,xyy}}^{j}+\sin2\beta_{_{i}}^{j}f_{_{,yyy}}^{j}\right)\right. \\ -D_{n}^{j}f_{_{,xx}}^{j}\left.+D_{n}\sin2\beta_{_{i}}^{j}y^{j}f_{_{,xyy}}^{j}-D_{n}\cos2\beta_{_{i}}^{j}y^{j}f_{_{,yyy}}^{j}\right] \end{matrix}$ (A-4)

$\begin{matrix} \sigma_{s}=2G\left[-\sin2\beta_{_{i}}^{j}D_{s}^{j}f_{_{,xy}}^{j}-\cos2\beta_{_{i}}^{j}D_{s}^{j}f_{_{,xx}}^{j}-D_{s}^{j}y^{j}\left(\sin2\beta_{_{i}}^{j}f_{_{,xyy}}^{j}-\cos2\beta_{_{i}}^{j}f_{_{,yyy}}^{j}\right)\right. \\ \left.-D_{n}^{j}\cos2\beta_{_{i}}^{j}y^{j}f_{_{,xyy}}^{j}-D_{n}^{j}\sin2\beta_{_{i}}^{j}y^{j}f_{_{,yyy}}^{j}\right] \end{matrix}$ (A-5)

Matrix expression of Eq. (A-5):

$\left[\begin{matrix} \sigma_{n} \\ \sigma_{s} \end{matrix}\right]=2G\left[\begin{matrix} 2\sin^{2}\beta_{_{i}}^{j}f_{_{,xy}}^{j}+2\sin2\beta_{_{i}}^{j}f_{_{,xx}}^{j}-\cos2\beta_{_{i}}^{j}y^{j}f_{_{,xyy}}^{j}-\sin2\beta_{_{i}}^{j}y^{j}f_{_{,yyy}}^{j} & -f_{_{,xx}}^{j}+\sin2\beta_{_{i}}^{j}y^{j}f_{_{,xyy}}^{j}-\cos2\beta_{_{i}}^{j}y^{j}f_{_{,yyy}}^{j} \\ -\sin2\beta_{_{i}}^{j}f_{_{,xy}}^{j}-\cos2\beta_{_{i}}^{j}f_{_{,xx}}^{j}-\sin2\beta_{_{i}}^{j}y^{j}f_{_{,xyy}}^{j}+\cos2\beta_{_{i}}^{j}y^{j}f_{_{,yyy}}^{j} & -\cos2\beta_{_{i}}^{j}y^{j}f_{_{,xyy}}^{j}-\sin2\beta_{_{i}}^{j}y^{j}f_{_{,yyy}}^{j} \end{matrix}\right]\left[\begin{matrix} D_{s}^{j} \\ D_{n}^{j} \end{matrix}\right]$ (A-6)

Where,

$A=2G\left[\begin{matrix} 2\sin^{2}\beta_{_{i}}^{j}f_{_{,xy}}^{j}+2\sin2\beta_{_{i}}^{j}f_{_{,xx}}^{j}-\cos2\beta_{_{i}}^{j}y^{j}f_{_{,xyy}}^{j}-\sin2\beta_{_{i}}^{j}y^{j}f_{_{,yyy}}^{j} & -f_{_{,xx}}^{j}+\sin2\beta_{_{i}}^{j}y^{j}f_{_{,xyy}}^{j}-\cos2\beta_{_{i}}^{j}y^{j}f_{_{,yyy}}^{j} \\ -\sin2\beta_{_{i}}^{j}f_{_{,xy}}^{j}-\cos2\beta_{_{i}}^{j}f_{_{,xx}}^{j}-\sin2\beta_{_{i}}^{j}y^{j}f_{_{,xyy}}^{j}+\cos2\beta_{_{i}}^{j}y^{j}f_{_{,yyy}}^{j} & -\cos2\beta_{_{i}}^{j}y^{j}f_{_{,xyy}}^{j}-\sin2\beta_{_{i}}^{j}y^{j}f_{_{,yyy}}^{j} \end{matrix}\right]$ (A-7)

Where, $\theta_{i}$is the included angle between unit *i* and the original coordinate system (geodetic coordinate system); $\beta_{_{i}}^{j}$is the included angle between unit *j* and unit *i*.

According to the superposition principle, the displacement and stress of the fault plane are superimposed by the action of all element discontinuities, and the tangential and normal stresses generated by the displacement discontinuities $D_{n}^{j}$and $D_{s}^{j}$ ($j=1,2,\cdots n$) of each element on element *i* are.

$\begin{matrix} \sigma_{s}^{i}=\sum_{j=1}^{n} A_{ss}^{ij}D_{s}^{j}+\sum_{j=1}^{n} A_{sn}^{ij}D_{n}^{j} \\ \sigma_{n}^{i}=\sum_{j=1}^{n} A_{ns}^{ij}D_{s}^{j}+\sum_{j=1}^{n} A_{nn}^{ij}D_{n}^{j} \end{matrix}$ (A-9)

Where, $A_{ss}^{ij}$, $A_{sn}^{ij}$ are the tangent constant displacement discontinuity of element *j* and the coefficient of shear stress and normal stress at the midpoint of element *i*; and $A_{ns}^{ij}$, $A_{nn}^{ij}$ are the normal constant displacement discontinuity of element *j* and the coefficient of shear stress and normal stress at the midpoint of element *i,* $MPa\bullet m^{-1}$.

The stress boundary conditions of the discontinuity plane during the fault slip process are:

$\begin{matrix} \sigma_{s}^{i}=\sigma_{\infty}-\Delta\sigma_{s}^{i} \\ \sigma_{n}^{i}=\sigma_{\infty}-p_{f}-\Delta\sigma_{n}^{i} \end{matrix}$ (A-10)

Substitute Eq. (A-10) into Eq. (A-9):

$\begin{matrix} \sigma_{\infty}-\Delta\sigma_{s}^{i}=\sum_{j=1}^{n} A_{ss}^{ij}D_{s}^{j}+\sum_{j=1}^{n} A_{sn}^{ij}D_{n}^{j} \\ \sigma_{\infty}-p_{f}-\Delta\sigma_{n}^{i}=\sum_{j=1}^{n} A_{ns}^{ij}D_{s}^{j}+\sum_{j=1}^{n} A_{nn}^{ij}D_{n}^{j} \end{matrix}$ (A-11)

There are 2n algebraic equations in Eq. (A-11), which are the same as the number of unknowns and can be solved by Newton’s iterative method.

The above assumed condition is a two-dimensional plane body, which means that the influence of vertical direction is ignored, which affects the actual calculation accuracy. Olson (2004) introduced a three-dimensional correction factor to account for the finite height of the fault plane. Eq. (A-11) is modified as：

$\begin{matrix} \sigma_{\infty}-\Delta\sigma_{s}^{i}=\sum_{j=1}^{n} G^{ij}A_{ss}^{ij}D_{s}^{j}+\sum_{j=1}^{n} G^{ij}A_{sn}^{ij}D_{n}^{j} \\ \sigma_{\infty}-p_{f}-\Delta\sigma_{n}^{i}=\sum_{j=1}^{n} G^{ij}A_{ns}^{ij}D_{s}^{j}+\sum_{j=1}^{n} G^{ij}A_{nn}^{ij}D_{n}^{j} \end{matrix}$ (A-12)

Where,

$G=1-\frac{d_{ij}^{\chi}}{\left[d_{ij}^{2}+\left(h/\delta\right)^{2}\right]^{\chi/2}}$ (A-13)

Where, $d_{ij}$is the distance between unit centers, m; *h* is the height of the fault plane, m; $\chi$, $\delta$ are dimensionless coefficients related to the fault area.

**Appendix B**

In the dynamic slip model of fault, the dynamic equations of Eqs. (A-9)-(A-13) are time dependent and must be solved iteratively. The dynamic explicit method does not require balance iteration, has a fast calculation speed, and can be guaranteed accuracy as long as the step size is small enough. However, when calculating the complex matrix, it is easy to trigger the hourglass mode, which affects the stress solution’s accuracy. In a large time step, the implicit algorithm can solve the complex matrix (static equilibrium equations) accurately. Based on this, the model’s solution combines the benefits of the two methods to introduce the dynamic explicit-static implicit coupling solution.

Firstly, to update and calculate the fault plane state variables and formation pressure, the explicit iteration (Runge-Kutta scheme) (Abramowitz and Stegun, 1972) is used. In this time step, the state variables, dynamic friction coefficient and accumulated slip D are all updated iteratively. In addition, during this iteration, the velocity v of each element can be calculated using the fault motion equation. In the second step, the implicit Euler coupling method Newton-Raphson iteration is used to solve the stress drop and stress value of the element body. The specific coupling iterative solution process is shown in Fig. B-1.

**Fig. B-1 The solving process coupling dynamic explicit-static implicit**

The time step adopts the adaptive adjustment mode, that is, according to the constraint conditions, the time is continuously adjusted to ensure accuracy. In this model, the constraint conditions mainly include: (1) error setting for explicit iteration of fault plane state variables $\psi$; (2) The change rate of formation pressure p; (3) The amplitude of acceleration change in each cell.
